# Phenolic Compounds with Antimicrobial Properties in Mushrooms Frequently Encountered in Temperate Deciduous Forests

**DOI:** 10.3390/life15111653

**Published:** 2025-10-23

**Authors:** Aida Puia, Stanca-Lucia Pandrea, Jeanine Cruceru, Ion Cosmin Puia, Veronica Sanda Chedea, Călina Ciont, Oana Lelia Pop, Loredana Florina Leopold, Floricuța Ranga, Adriana Cristina Urcan, Alexandru Nicolescu, Otilia Bobis, Ioana Corina Bocsan, Sebastian Armean, Anca Dana Buzoianu, Raluca Maria Pop

**Affiliations:** 1 Discipline of Family Medicine, Department Community Medicine, Faculty of Medicine, ”Iuliu Hatieganu” University of Medicine and Pharmacy, 19 Moţilor Street, 400347 Cluj-Napoca, Romania; aida.puia@umfcluj.ro; 2 Fundamental Disciplines, Microbiology, Department 1, “Iuliu Hatieganu” University of Medicine and Pharmacy, 5 Pasteur Street, 400349 Cluj-Napoca, Romania; 3 Medical Analysis Laboratory, “Octavian Fodor” Regional Institute of Gastroenterology and Hepatology, 19-21 Croitorilor Street, 400162 Cluj-Napoca, Romania; 4 Pharmacology, Toxicology and Clinical Pharmacology, Department of Morphofunctional Sciences, “Iuliu Haţieganu” University of Medicine and Pharmacy, Victor Babeș, No 8, 400012 Cluj-Napoca, Romania; crucerujeanine@gmail.com (J.C.); bocsan.corina@umfcluj.ro (I.C.B.); sebastian.armean@umfcluj.ro (S.A.); abuzoianu@umfcluj.ro (A.D.B.); raluca.pop@umfcluj.ro (R.M.P.); 5 Department of Surgery, Faculty of Medicine, “Iuliu Hatieganu” University of Medicine and Pharmacy, 19-21 Croitorilor Street, 400347 Cluj-Napoca, Romania; 6 “Octavian Fodor” Regional Institute of Gastroenterology and Hepatology, 3rd General Surgery Clinic, 19-21 Croitorilor Street, 400162 Cluj-Napoca, Romania; 7 Research Station for Viticulture and Enology Blaj (SCDVV Blaj), 515400 Blaj, Romania; chedeaveronica@yahoo.com; 8 Faculty of Food Science and Technology, University of Agricultural Sciences and Veterinary Medicine, 3–5 Calea Mănăștur, 400372 Cluj-Napoca, Romania; calina.ciont@usamvcluj.ro (C.C.); oana.pop@usamvcluj.ro (O.L.P.); loredana.leopold@usamvcluj.ro (L.F.L.); florica.ranga@usamvcluj.ro (F.R.); 9 Molecular Nutrition and Proteomics Laboratory, Institute of Life Sciences, University of Agricultural Sciences and Veterinary Medicine, 400372 Cluj-Napoca, Romania; 10 Department of Microbiology and Immunology, Faculty of Animal Science and Biotechnology, University of Agricultural Sciences and Veterinary Medicine, 400372 Cluj-Napoca, Romania; adriana.urcan@usamvcluj.ro; 11 Laboratory of Chromatography, Faculty of Horticulture and Business in Rural Development, University of Agricultural Sciences and Veterinary Medicine, 400372 Cluj-Napoca, Romania; alexandru.nicolescu@usamvcluj.ro; 12 Department of Apiculture and Sericulture, Faculty of Animal Science and Biotechnology, University of Agricultural Sciences and Veterinary Medicine Cluj-Napoca, 400372 Cluj-Napoca, Romania; obobis@usamvcluj.ro

**Keywords:** *Cantharellus cibarius*, *Russula virescens*, *Lactarius piperatus*, *Boletus edulis*, phenolic compounds, antioxidant activity, antimicrobial activity, antibacterial activity

## Abstract

Mushrooms have long been recognized as a rich source of bioactive compounds, including phenolics, that possess important antioxidant, antimicrobial, and antibacterial properties, including activity against drug-resistant bacteria. This study evaluated total phenolic profile and content, total flavonoids content, the antioxidant activities, antimicrobial and antibacterial activities, of water extracts of edible mushrooms from Romanian deciduous forests, including *Cantharellus cibarius*, *Russula virescens*, *Lactarius piperatus*, and *Boletus edulis*. The extracts were characterized using high-performance liquid chromatography coupled with mass spectrometry (HPLC-MS) and Fourier Transform Infrared Spectroscopy (FTIR) analysis. Antioxidant activity was determined using DPPH radical-scavenging activity and ABTS radical cation decolorization assay. Antimicrobial and antibacterial activities were tested using standard strains of *Staphylococcus aureus*, *Escherichia coli*, *Pseudomonas aeruginosa*, *Enterococcus faecalis*, and *Streptococcus pneumoniae* following diffusion testing and time-killing assay, respectively. The HPLC-MS results indicated that major compounds in all the mushrooms belonged to the subclass of hydroxybenzoic acids. Trans-cinnamic acid and hydroxybenzoic acids, particularly gallic acid, 2,3-dihydroxybenzoic acid, and gentisic acid, were the predominant compounds detected in BEE and CCE. Their concentrations were measured as follows: 24 μg/mL, 63 μg/mL, 56 μg/mL, and 14 μg/mL, respectively, for BEE, and 26 μg/mL, 42 μg/mL, 7 μg/mL, and 5 μg/mL, respectively, for CCE. Among phenolic compounds, 2-dihydroxybenzoic acid, 2,3-dihydroxybenzoic acid, *p*-anisaldehyde, and gentisic acid were positively correlated with both DPPH (45% and 21% inhibition rate for BEE and CCE, respectively) and ABTS (64 and 31% inhibition rate for BEE and CCE, respectively) antioxidant activities. The FTIR analysis revealed the presence of lipids, proteins, and polysaccharides, extracted in different ratios in the water extract. All mushroom extracts showed a dose-dependent response with higher antimicrobial and antibacterial activities at the highest concentration (26.3 µg phenolics BEE, 12.7 µg pphenolics CCE, 28.3 µg phenolics LPE, and 14.5 µg phenolics RVE per well for antimicrobial activity and 175.2 µg phenolics/mL BEE, 84.4 µg phenolics/mL CCE, and 188.9 µg phenolics/mL LPE for antibacterial activity). These species demonstrate potential for the development of alternative antimicrobial formulations, particularly relevant in the context of antibiotic resistance.

## 1. Introduction

Mushrooms have a longstanding history of being used as both a nutritional and therapeutic source, especially within the continent of Asia. They represent a great source of protein, fiber, carbohydrates, vitamins, and minerals and are also suitable for the majority of diets due to their low-fat and low-calorie profiles [1]. Species like *Cantharellus cibarus* (*C. cibarius*) and *Rusulla virescens* (*R. virescens*), which are commonly found across different climates, have been reportedly used by tribes in India both for feeding purposes and as medicine for a long time, for helping women with their labor and delivery, respectively, and for treating wounds, representing one of the main sources of natural medicine [2].

Their widespread availability and their nutritional content make mushrooms nowadays one of the primary food sources and a means of earning income all around the world [3]. With the pressing issues imposed by climatic changes and their impact on agriculture and food sourcing, mushrooms stand out as a factor that could mitigate the effect these environmental issues have had on the food supply worldwide [3]. Furthermore, the crucial role of mushrooms is not limited to being an important nutritional source, and their implication in the decomposition of organic matter can aid plant growth, thus representing an important link in the cycle of nutrients and carbon [4].

As far back as Fleming’s discovery of penicillin, the first antibiotic with large-scale applications in 1929, multiple studies underlined the potential properties of fungi-derived compounds. Fungi have adapted to the most unfavorable environments in which they are competing against other organisms for survival by producing certain secondary metabolites that can be of great importance and can represent innovative remedies in modern-day medicine [5].

Mushrooms are a unique source of antioxidant-rich food products since they are naturally associated with antimicrobial properties. They are abundant in polyphenols and flavonoid derivatives, which studies have proven to activate similar cellular pathways that are normally involved in countering oxidative stress [6]. Epidemiologic studies have shown that there is a correlation between a diet high in phenolic compounds and lower incidences of certain health issues, such as cardiovascular diseases, strokes, or even certain types of cancer, which can be attributed to their anti-inflammatory, antioxidant, and antibacterial properties [7].

There are reports regarding antimicrobial effects that date as far back as the 1940s, with multiple antimicrobials like grifolin, ganomycins, pleuromutilin, and others being isolated from the filamentous fungi that make up the *Basidiomycota phylum* [5]. Moreover, several genera of medicinal mushrooms, including *Pleurotus* and *Ganoderma*, have been noted as having antimicrobial activity against drug-resistant forms of *Staphylococcus* or *Escherichia coli* [8]. Because of their widespread growth area, out of which the better part of it is naturally occurring, mushrooms could offer an ideal substitute to first-choice antibiotics against which there has been an increased prevalence of antibiotic resistance among bacteria [9].

The widespread forest areas that can be found nationally in Romania allow for species of mushrooms such as *C. cibarius* and *Boletus edulis* (*B. edulis*) to gain a significant economic importance beyond their main role as a food source for the locals. Ample studies cover the medicinal applications of both species [10,11], which only raises the value of the species as a sought-after item when it comes to the global trade [12], making it one of the main sources of income for rural communities. The interdependence with their ecosystems makes them very difficult to cultivate artificially, which poses a challenge for widespread commercialization and, in turn, raises their market value [13,14]. Species like *R. virescens* have applications beyond their nutraceutical and dietary roles, with its extract being regarded as an important component of many anti-aging cosmetics for its antioxidant properties [15]. Unlike the mushrooms mentioned before, *Lactarius piperatus* (*L. piperatus*) cannot be considered an important food source for the communities around its ecosystem, though it can be used for seasoning purposes due to its spicy taste. However, its primary application lies in biotechnology and agriculture as a biosorbant for heavy metals in contaminated waters and soil [16] and a promoter of plant growth through auxins [17].

While many studies focus on determining the total phenolic content of different species of mushrooms, not many of them focus on identifying the individual phenols that can be found in their fruiting bodies [9,18,19,20]. This study aimed to assess the total phenolic and flavonoid contents, the antioxidant activities, as well as the antimicrobial and antibacterial activities, of mushroom species commonly harvested for consumption from deciduous forests in Romania—such as *C. cibarius*, *R. virescens*, *L. piperatus*, and *B. edulis*—while also providing a comprehensive profile of their specific phenolics.

## 2. Materials and Methods

### 2.1. Chemicals

Sodium carbonate (Na_2_CO_3_), 2,2′-azino-bis (3-ethylbenzothiazoline-6-sulfonic acid (ABTS), potassium persulfate, sodium nitrite (NaNO_2_), methanol, ethanol, distilled water, aluminum trichloride (AlCl_3_), sodium hydroxide (NaOH), zinc selenide, acetone, acetic acid, gallic acid, acetone, and 2,2-diphenyl-1-picrylhydrazyl (DPPH) were purchased from Merck Co. (Darmstadt, Germany). All ultra-pure grade chemicals, as well as the standard solution for the HPLC analysis, were purchased from Sigma Co. (St. Louis, MO, USA) and Merck Co. (Darmstadt, Germany). Thus, phenolic standards of chlorogenic acid (≥95% purity, Sigma-Aldrich, St. Louis, MO, USA, Lot 032K1336), luteolin (≥99% purity, Fluka, Industriestrasse 25, CH-9471 Buchs/Switzerland, Lot WA18442), rutin (≥94% purity, Sigma-Aldrich, Lot #BCBD8327V), and gallic acid (Sigma-Aldrich, Lot #BCCC0553) were used for calibration. The HPLC-grade acetonitrile was sourced from Merck (Darmstadt, Germany), while ultrapure water was obtained with the Direct-Q UV system (Millipore, Burlington, MA, USA).

### 2.2. Plant Material

The species of mushrooms analyzed in this study were *C. cibarius* (Cantharellaceae)*, R. virescens* (Russulaceae), *L. piperatus* (Russulaceae), and *B. edulis* (Boletaceae). Fresh fruiting bodies from all four species were harvested by professional gatherers from the area of the Meseș hill, close to Zalau (47°07′ N 23°02′ E, 780–820 m a.s.l.), Romania, in September 2024. The collection sites were located in mixed deciduous–coniferous forests dominated by *Fagus sylvatica* (European beech) and *Picea abies* (Norway spruce). The average temperature and relative humidity during collection were approximately 16 °C and 78%, respectively. Specimens were gathered from soil substrates covered with leaf litter under shaded canopy conditions. Morphological identification was performed based on macroscopic features, including cap shape, color, and surface texture; gill attachment and spacing; stipe morphology; latex presence (for *Lactarius*); and spore print color. We chose these species because they are easy to identify and large quantities are consumed every year, usually from the local markets. Afterwards, mushrooms were identified by Prof. dr. Marcel Pârvu from the “Babeș-Bolyai” Cluj-Napoca University, Biology Department. The taxonomical classification was carried out at the University of Agricultural Science and Veterinary Medicine in Cluj-Napoca, Romania.

### 2.3. Extraction Protocol

The freshly collected fruiting bodies of the mushrooms, weighing 200 g for each species, were finely ground up and immediately mixed with 400 mL of distilled water. The mixtures were sonicated in a Witeg WiseClean WUC-D06H ultrasonic bath (FilaraBiomed, Cluj-Napoca, Romania) for 40 min (ultrasonic power of 200 W at 50 °C) before being filtered and subjected to evaporation with a vacuum rotary evaporator at 40 °C (Hei-VAP Advantage, Heidolph, Nitech Cluj-Napoca, Romania). Four mushroom concentrated extracts: BEE, from *B. edulis*, LPE from *L. piperatus*, RVE from *R. virescens*, and CCE from *C. cibarius* were obtained and used for the subsequent measurements.

### 2.4. Total Phenolic Content

The Folin–Ciocalteu method was employed to determine the total phenolic content (TPC), similar to a previous study [21,22]. A volume of 25 μL of the mushroom extract was mixed with 125 μL of Folin–Ciocalteu reagent (0.2 N), as well as 100 μL of sodium carbonate (Na_2_CO_3_) solution (7.5% *w*/*v*). The blend was incubated in the dark, at room temperature for 2h. Absorbance was measured with a Synergy HT Multi-Detection Microplate Reader with 96-well plates (BioTek Instruments, Inc., Winooski, VT, USA), at 760 nm, using Gallic acid as a standard (r^2^ = 0.9945). Thus, the results were expressed as mg of gallic acid equivalents (GAE) per gram of fresh mushroom weight (FW). All extracts were analyzed in triplicate, the results being expressed as the mean values with the standard deviation associated.

### 2.5. Total Flavonoid Content

The total flavonoid content (TFC) was determined based on the interaction between flavonoids and aluminum trichloride [23]. In brief, 1 mL of each mushroom extract was mixed with 0.3 mL NaNO_2_ 5%, 0.3 mL AlCl_3_ 10% and 2 mL NaOH solution (1 M). The volume of the mixture obtained was aliquoted to 10 mL using distilled water. Following a 15 min incubation time, the maximum absorbance was read at 510 nm using a Jasco V-530 spectrophotometer. Quercetin represented the calibration standard (r^2^ = 0.9914). The TFC was measured in mg of quercetin equivalents (QE) per 100 g fresh weight. All extracts were analyzed in triplicate, the results being expressed as the mean values with the standard deviation associated.

### 2.6. DPPH Radical-Scavenging Activity

To determine the DPPH radical-scavenging activity of mushrooms, a slightly modified version of Brand-Williams et al. was used [24]. In a ratio of 1:7, a volume of 250 μL of sample was mixed with 1750 μL DPPH solution (0.02 mg/mL in methanol). The mixture was incubated at room temperature for half an hour, and the absorbance was measured at 517 nm with a Synergy HT Multi-Detection Microplate Reader with 96-well plates (BioTek Instruments, Inc., Winooski, VT, USA). The formula used to calculate the inhibition percentage is as follows:

$$DPPH\%=\frac{Ac-As}{Ac}\;\;\times 100$$

where Ac = the absorbance of the control and As = absorbance of the sample.

### 2.7. ABTS Radical Cation Decolorization Assay (ABTS+)

The antioxidant activity of the mushrooms was determined using the modified procedure previously described [25,26]. Mixing a 7 mM aqueous solution of ABTS+ and 2.45 mM potassium persulfate, we obtained a blue green ABTS+ solution that was kept at room temperature in the dark for 12 to 16 h before use, allowing the reaction to occur. The working ABTS+ solution was obtained by diluting the stock solution with ethanol for an absorbance of 0.700 ± 0.02 AU at 734 nm. Different concentrations of the mushroom extracts were combined with 20 µL Trolox and 170 µL ABTS+ solutions, all of which were incubated in the dark for 6 min at room temperature. Their absorbance was measured using a microplate reader (BioTek Instruments, Winooski, VT, USA) and used to express the antioxidant activity using the formula:

$$ABTS\%=\frac{Ac-As}{Ac}\;\times \;100$$

where Ac = the absorbance of the control and As = absorbance of the sample.

### 2.8. Fourier Transform Infrared Spectroscopy (FTIR) Analysis

To record the FTIR spectra of the mushroom extracts, we used a Zinc Selenide (ZnSe) composite internal reflection accessory and an FTIR spectrometer Shimadzu IR Prestige- 21 with included attenuated total reflectance (ATR). With the extracts being directly applied to the ATR crystal, 64 scans were performed for each spectrum ranging from 4000 to 650 cm^−1^, with the air spectrum as a background. All measurements were made following a thorough cleaning of the ATR crystal with acetone.

### 2.9. Liquid Chromatography-Diode Array Detection–Electro-Spray Ionization Mass Spectrometry (HPLC-DAD-ESI MS)

For this method of characterizing the mushrooms extracts, an Agilent 1200 HPLC system (Agilent Technologies, Santa Clara, CA, USA), equipped detector with photodiode array (DAD) and a single quadrupole mass spectrometer (MS) detector, model 6110, was utilized [17]. The compound separation took place on a Kinetex XB C18 column (4.6 × 150 mm, 5 μm particle size; Phenomenex, Torrance, CA, USA). There were two mobile phases used, out of which the first one was an aqueous solution with 0.1% acetic acid (mobile phase A) and the second was obtained from mixing acetonitrile with 0.1% acetic acid (mobile phase B). The duration of each run was 30 min. Temperature was set at 25 °C, with a flow rate of 0.5 mL/min. The first phase of the elution program started at 0 min with 5%B and lasted two minutes. Following that, the concentration gradually increased from 5% to 40%B until reaching the 18 min mark. From 18 to 20 min, the concentration was further increased up to 90% B and maintained at that concentration for 4 min. From minute 24 to minute 25, the concentration was rapidly decreased back to 5%B, where it was maintained for the last 5 min of the 30 min run. The spectral data for all peaks was collected in the 200–600 nm range while the chromatograms were recorded at 280 and 340 nm wavelengths. The MS analysis was achieved using an ESI positive full scan ionization mode, with a capillary voltage of 3000 V, a temperature of 350 °C, a nitrogen flow of 7 L/min, and a mass range between 120 and 1200 *m*/*z*. Acquisition and interpretation of the data obtained was carried out using Agilent ChemStation software, version B.02.01-SR2 [17]. The calibration curves for quantifying phenolic compounds were achieved by injecting five different concentrations of each standard dissolved in methanol. These calibration curves produced the equations further used for the quantitative analysis of each phenolic compound (*n* = 3), as follows: hydroxycinnamic acids were quantified as chlorogenic acid equivalents (y = 22.585x − 36.728, R^2^ = 0.9937, LOD = 0.41 μg/mL, LOQ = 1.24 μg/mL); flavones as luteolin equivalents (y = 68.857x + 25.113, R^2^ = 0.9972, LOD = 0.38 μg/mL, LOQ = 1.14 μg/mL); flavonols as rutin equivalents (y = 26.935x − 33.784, R^2^ = 0.9981, LOD = 0.21 μg/mL, LOQ = 0.64 μg/mL) and hydroxybenzoic acids as gallic acid equivalents (y = 33.624x + 30.8; R^2^ = 0.9978; LOD = 0.35 μg/mL, LOQ = 1.05 μg/mL). The mass spectra, retention period, and UV-Vis absorption were matched against reference standards, previous published data, and information from the Phenol-Explorer database to ensure a proper characterization of the phenolic compounds in the mushroom extracts.

### 2.10. Antimicrobial Activity

The water extracts were dissolved in dimethyl sulfoxide (DMSO) in a 1:1 ratio (0.5 mL of extract and 0.5 mL of DMSO) and filter-sterilized through a 0.45-μm membrane filter. The concentration of the extracts was calculated considering the total phenolic content as determined by HPLC-MS analysis and presented in Section 3.4. The antimicrobial activity of mushroom extracts (BEE, LPE, RVE, and CCE) was determined using the agar well diffusion method [27]. Standard strains of *Staphylococcus aureus* ATCC 29213 (*S. aureus*), *Escherichia coli* ATCC 25922 (*E. coli*), *Pseudomonas aeruginosa* ATCC 27853 (*P. aeruginosa*), *Enterococcus faecalis* ATCC 29212 (*E. faecalis*), and *Streptococcus pneumoniae* ATCC 49619 (*S. pneumoniae*) cultures were used. Each bacterial strain was cultured on blood agar for 24 h. *S. pneumoniae* ATCC 49619 was incubated in a 5% CO_2_ atmosphere. Bacterial suspensions were then prepared in normal saline solution and adjusted to a turbidity of 0.5 McFarland, corresponding to an approximate concentration of 1–2 × 10^8^ CFU/mL. These strains were selected since they are the most frequent causes of nosocomial infections and resistance to antibiotics.

Diffusion testing: 0.5 McFarland suspension of each bacterial strain was inoculated onto Mueller-Hinton agar plate (bioMérieux SA, Marcy-l’Étoile, France) using the streak method with a sterile swab, except *S. pneumoniae* ATCC 49619, which was cultured onto Mueller-Hinton agar with 5% defibrinated horse blood and 20 mg/mL ß-NAD (MH-F agar, bioMérieux SA, Marcy-l’Étoile, France). After inoculation, sterile wells were created. One hundred microliters (BEE, LPE, RVE, CCE) and 150 microliters (BEE, LPE, RVE, CCE) of each extract of mushrooms were loaded into the different wells. Ampicillin (10 μg/disk) for bacteria was used as a positive control, and DMSO was used as a negative control for the tested microorganisms. The plates were incubated for 24 h at 37 °C. After the incubation period, the diameter of the inhibition zone was measured in millimeters. All the tests were carried out in triplicate, and their means were recorded.

### 2.11. Time-Kill Assay

The time-kill assay was performed to evaluate the antibacterial activity of the tested mushroom extracts. The following bacterial strains were used: *E. faecalis* (ATCC 29212), *E. coli* (ATCC 25922), *P. aeruginosa* (ATCC 27853), *S. aureus* (ATCC 25923), and *S. pneumoniae* (ATCC 49619). Bacterial suspensions were adjusted to a final concentration of approximately 10^6^ CFU/mL in Mueller–Hinton broth. The assay was conducted in sterile 96-well microplates by mixing 10 µL of each bacterial suspension with various concentrations of the extract, previously prepared as described, and diluted in DMSO (50%, 25%, 10%, 5%, 2.5%, and 1.25% *v*/*v*). The concentration of the extracts was calculated considering the total phenolic content as determined by HPLC-MS analysis and presented in Section 3.5. The plates were incubated at 37 °C for 24 h. Bacterial growth was assessed by measuring optical density at 600 nm (OD_600_) using a BioTek Synergy 2 multimode microplate reader (BioTek Instruments, Winooski, VT, USA). Positive controls consisted of bacterial suspensions in broth without extract, while negative controls contained broth only. To allow comparison between conditions, for each time point, the OD_600_ value of the extract-treated sample was divided by the OD_600_ of the positive control (bacteria without extract), allowing relative bacterial growth to be calculated. All measurements were performed in triplicate.

### 2.12. Statistical Analysis

For the statistical analyses, three biological replicates were used. Statistical analysis was performed using SPSS version 29.0.0.0 (IBM Corp., Armonk, NY, USA). Data distribution was checked using the Shapiro–Wilk test. Data showing normal distribution were presented as mean ± standard deviation, while the ones with non-normal distribution were presented as medians and interquartile ranges (25th–75th percentiles). For normally distributed data, comparison was performed using one-way analysis of variance (ANOVA), while for the non-parametric data, the Kruskal–Wallis test was used. Statistical significance was considered at *p* < 0.05. Principal component analysis (PCA) was performed using Unscrambler (v10.1, CAMO Software AS, Oslo, Norway) to examine sample clustering. Pearson correlation was calculated in SPSS, and heatmaps based on Pearson correlation were generated with Orange Data Mining (v3.39.0, University of Ljubljana, Slovenia).

## 3. Results

### 3.1. Total Polyphenols, Flavonoids, and Antioxidant Activities of Mushroom Extracts

TPC, TFC, and antioxidant activity as determined by DPPH and ABTS tests of the four mushroom water extracts are presented in Table 1.

The highest antioxidant DPPH activity was registered for the BEE extract (45%), significantly different from the others. *Russula virescens* and CCEs had almost half of the BEE DPPH inhibition rate and were not statistically different from each other. Concerning the ABTS radical scavenging assay, BEE presented significantly higher values (64%) as compared with other extracts, which had approximately half of its radical scavenging properties (Table 1). The Pearson correlation showed a significant correlation between DPPH and ABTS antioxidant capacities, but did not show any significant correlation between them and TPC or TFC. As observed, LPE had the highest TPC (201 mg GAE/100 g FW) among mushroom extracts but lower DPPH (25%) and ABTS (31%) values. As far as the total flavonoid content is concerned, the highest content was measured for the LPE (6 mg QE/100 g FW) extract, while RVE had the lowest content (5 mg QE/100 g FW).

### 3.2. Chemical Fingerprinting of Mushroom Extracts Using FTIR Analysis

**Figure 1:**
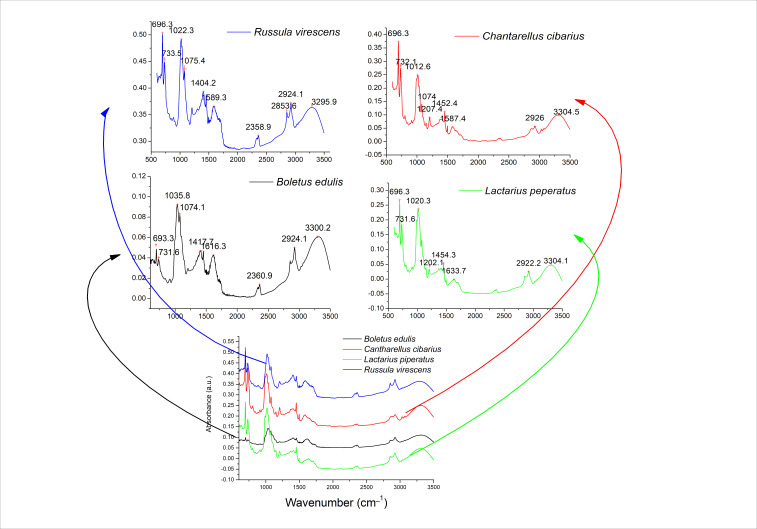
FTIR mushroom water extracts recorded between 3500 and 600 cm^−1^.

Figure 1 presents the comparison of FTIR spectra of water mushroom extracts recorded between 3500 and 600 cm^−1^. The infrared absorption bands in the mushroom extracts were, in general, similar, with regions corresponding to the mushroom’s specific functional groups of their chemical composition. Absorption bands between 3500 and 3000 cm^−1^ can represent the presence of polysaccharide chains, water, or phenolic compounds in the mushroom extracts. The absorption bands in this region are mainly produced by the stretching O-H vibration present in these molecules [28]. In this sense, high-intensity bands at 3296, 3305, 3300, and 3304 were present in RVE, BEE, CCE, and LPE, respectively (Figure 1). The high-intensity bands between 2926 cm^−1^ and 2850 cm^−1^ were correlated to the -CH_2_ asymmetric and symmetric lipid stretching [29]. The absorption band at 2854 cm^−1^ from lipids and probably carbohydrates is more intense in RVE and BEE extracts than CCE and LPE extracts. Next, the high-intensity bands between 1650 and 1550 cm^−1^ can be attributed to the amide I or II bands in the proteins or peptides. Specifically, the absorption band at 1616 cm^−1^ (in BEE), 1634 cm^−1^ (in LPE), 1589 cm^−1^(in RVE), and 1587 cm^−1^ (in CCE) are distinctive for C=O stretching in proteins or glucans, N–H bending and C–N bending in proteins due to the amide I or amide II, vibrations [28]. The polysaccharide content was indicated through the C–C and C–O stretching vibrations in glycosidic bonds and pyranoid rings, which led to the presence of high-intensity bands within 1200–800 cm^−1^. Specifically, high-intensity bands at 1022 cm^−1^ (in RVE), 1012 cm^−1^ (in CCE), 1074 and 1036 cm^−1^ (in RVE and BEE) could be due to C-O-C and C-OH stretching vibrations in sugars, glucans, or chitin [30]. Finally, absorption bands between 800 and 600 cm^−1^ could be due to the out-of-plane bending in aromatic rings from phenolic compounds and flavonoids [30]. These results indicate that the mushroom extract contained phenolic compounds, lipids, proteins, and polysaccharides, extracted in different ratios from the mushroom, according to each variety. The bands identified in the mushroom extracts indicated similarities between RVE and BEE, with higher intensity bands for RVE. Also, CCE and LPE had similar fingerprints, with higher intensity bands observed for CCE.

**Figure 2:**
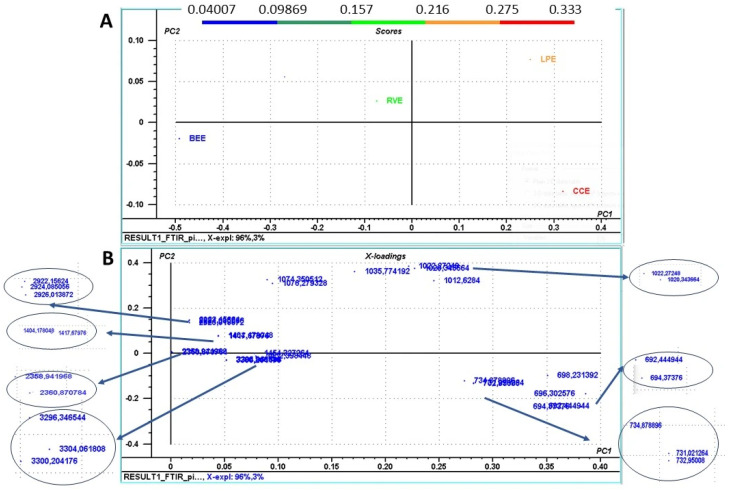
Principal component analysis (PCA) score plot of the first two principal components, PC1 and PC2 (**A**), of different peaks identified using Fourier transform infrared (FTIR) analysis in the mushroom extracts; (**B**) loading plot of PC1 and PC2 identifying the peak areas that is influencing mushroom samples distribution within the score plot; The peak areas used for PCA are: 692.44 cm^−1^; 694.37 cm^−1^; 696.30 cm^−1^; 698.23cm^−1^; 731.02 cm^−1^; 732.95 cm^−1^; 734.87 cm^−1^; 1012.62 cm^−1^; 1020.34 cm^−1^; 1022.27 cm^−1^; 1035.77 cm^−1^; 1074.35 cm^−1^; 1076.27 cm^−1^; 1404.17 cm^−1^, 1417.67 cm^−1^, 1452.39 cm^−1^; 2358.94 cm^−1^; 2360.87 cm^−1^; 2922.15 cm^−1^; 2924.08 cm^−1^; 2926.01 cm^−1^; 3296.34 cm^−1^; 3300.20 cm^−1^; and 3304.06 cm^−1^. The mushroom extracts were BEE (extract from *Boletus edulis*); LPE (extract from *Lactarius piperatus*); CCE (extract from *Cantharellus cibarius*); RVE (extract from *Russula virescens*). The blue arrows in the figure represents zoom sections.

Further, to be able to compare the proportional differences in the compounds identified by the FTIR analysis in the mushroom extracts, principal component analysis (PCA) multivariate analysis was perfomed (Figure 2). The analysis was performed using the absorbance peak intensities of all identified peaks. The PCA revealed the peaks that had the highest impact on mushroom discrimination among the PCA score plot (Figure 2A). According to Figure 2A, the variability in peak intensities was explained by the first two principal components (PCs), which explained 99% of the total sample variance, with a clear separation of mushrooms. The PCA loading plots (Figure 2B) showed that bands at 698 cm^−1^, 696 cm^−1^, 694 cm^−1^, 734 cm^−1,^ and 732 cm^−1^ significantly influenced mushroom sample clustering along the PC1 axis, while peaks at 1012 cm^−1^, 1020 cm^−1^, 1022 cm^−1^, and 1035 cm^−1^ influenced sample clustering along the PC2 axis. Correlating the PCA score plot with data represented in the loading plot, a positive correlation (higher area values of selected peaks) in the case of CCE and LPE, and a negative correlation (lower area values) specific to BEE was observed. As expected, the bands at the peaks between 800 and 600 cm^−1^ specific for phenolic compounds and flavonoids influenced the most sample clustering.

Also, absorption bands between 1012 and 1035 cm^−1^ corresponding to the stretching vibrations in sugars, glucans, or chitin influenced mushrooms separations, highlights their importance.

### 3.3. HPLC-DAD-ESI(+)MS-Based Profiling of Phenolic Constituents in Mushroom Extracts

**Figure 3:**
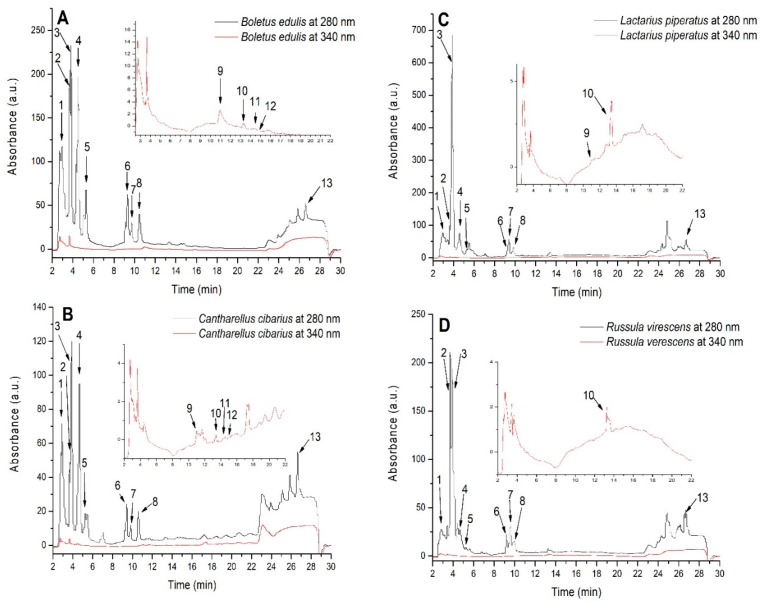
HPLC-MS chromatograms of *Boletus edulis* (**A**), *Cantharellus cibarius* (**B**), *Lactarius piperatus* (**C**), and *Russula virescens* (**D**) water extracts. Compound identification is presented in Table 2.

The analysis led to the detection of 13 compounds (Figure 3 and Table 2). Compounds belonging to hydroxybenzoic acids were the major compounds in all mushroom extracts. The highest concentration and percentage were recorded for LPE and BEE, with 90% of total polyphenols content as quantified by HPLC-MS, followed by RVE and CCE with 80%. Among hydroxybenzoic compounds, 3-dihydroxybenzoic acid was found in significantly higher concentration in LPE extract (170 μg/mL), followed by RVE, BEE, and CCE with 73, 60, and 37 μg/mL, respectively. The distribution of hydroxybenzoic acids was statistically different among mushroom varieties, and generally, BEE had the highest concentrations. Next, hydroxycinnamic acids were also present in lesser percentages, ranging from 15% in CCE to 9.5% in RVE and approximately 7% in BEE and LPE, respectively.

**Figure 4:**
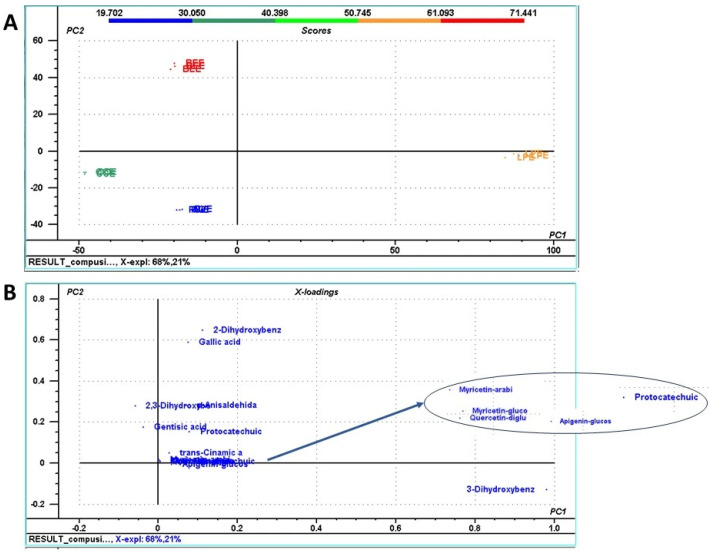
Principal component analysis (PCA) score plot of the first two principal components, PC1 and PC2 (**A**), of different peaks identified using High Performance Liquid Chromatography (HPLC) analysis in the mushroom extracts; (**B**) loading plot of PC1 and PC2 identifying the phenolic compounds that is influencing mushroom samples distribution within the score plot. The phenolic compounds used to generate the PCA score plot are: 2-dihydroxybenzoic acid; 2,3-dihydroxybenzoic acid; 3-dihydroxybenzoic acid; gallic acid; p-anisaldehyde; protocatechuic acid-glucoside; protocatechuic acid; gentisic acid; myricetin-arabinoside; apigenin-glucoside; myricetin-glucoside; quercetin-diglucoside; and trans-cinnamic acid. The mushroom extracts were: BEE (extract from *Boletus edulis*); LPE (extract from *Lactarius piperatus*); CCE (extract from *Cantharellus cibarius*); RVE (extract from *Russula virescens*).

Next, PCA was applied to the phenolic compounds as well, to identify phenolics that contributed most to sample differentiation. According to Figure 4A, the variability of phenolic compound concentration was explained by the first two principal components (PCs), which explained 89% of the total sample variance. The first principal component (PC1) explained 68% of the total variance, while the second one (PC2) explained the remaining 21%. The PCA loading plots (Figure 4B) showed that 3-dihydroxybenzoic acid significantly influenced mushroom sample clustering along the PC1 axis, while 2-dihydroxybenzoic acid, gallic acid, 2,3-dihydroxybenzoic acid, *p*-anisaldehyde, and gentisic acid influenced sample clustering along the PC2 axis. Correlating the PCA score plot with data represented in the loading plot, a positive correlation (higher concentration values) of 3-dihydroxybenzoic acid in the case of LPE, and a negative correlation specific to CCE was observed. As expected, compounds belonging to the hydroxybenzoic acid subclass, specific for mushroom extracts, influenced the most sample clustering.

**Figure 5:**
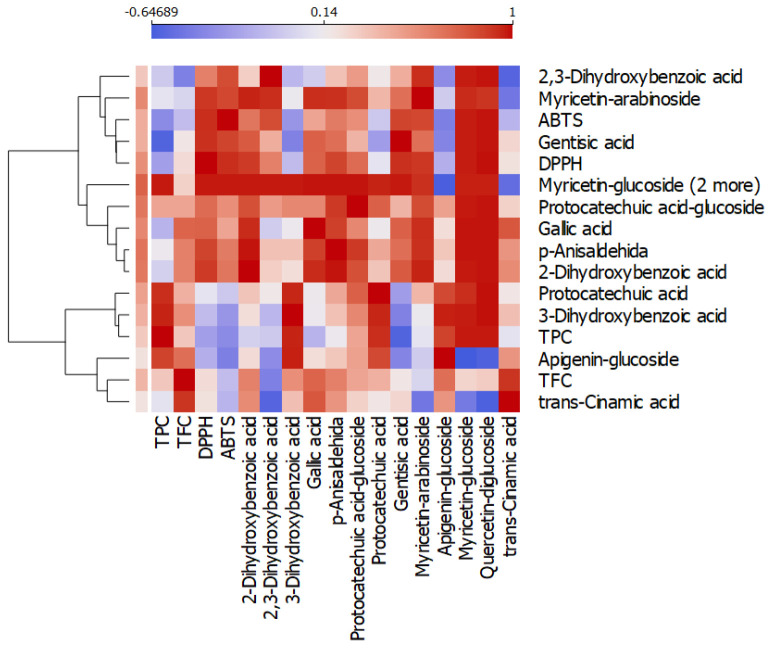
Heat map analysis, which visually encodes the Pearson correlation coefficients among the following variables: total phenolic compounds, total flavonoids, antioxidant DPPH and ABTS activities (see Table 1), and phenolic compounds (see Table 2), in the 4 mushroom extracts.

The Pearson correlation between antioxidant activities and phenolic compounds is reflected in Figure 5 and Table S2. Accordingly, TPC was positively correlated with 3-dihydroxybenzoic acid (*r* = 0.955, *p* < 0.001), protocatechuic acid (*r* = 0.929, *p* < 0.001), apigenin-glucoside (*r* = 0.871, *p* < 0.001), myricetin-glucoside (*r* = 0.974, *p* < 0.001), and quercetin-glucoside (*r* = 0.977, *p* < 0.001). Total flavonoid content was correlated with 2-dihydroxybenzoic acid (*r* = 0.620, *p* < 0.032), gallic acid (*r* = 0.739, *p* < 0.006), *p*-anisaldehyde (*r* = 0.623, *p* < 0.031), and apigenin-glucoside (*r* = 0.699, *p* < 0.011).

Among phenolic compounds, 2-dihydroxybenzoic acid, 2,3-dihydroxybenzoic acid, *p*-anisaldehyde, and gentisic acid were positively correlated with both DPPH and ABTS antioxidant activities (Table S2).

### 3.4. In Vitro Assessment of the Antimicrobial Efficacy of Mushroom Extracts

**Figure 6:**
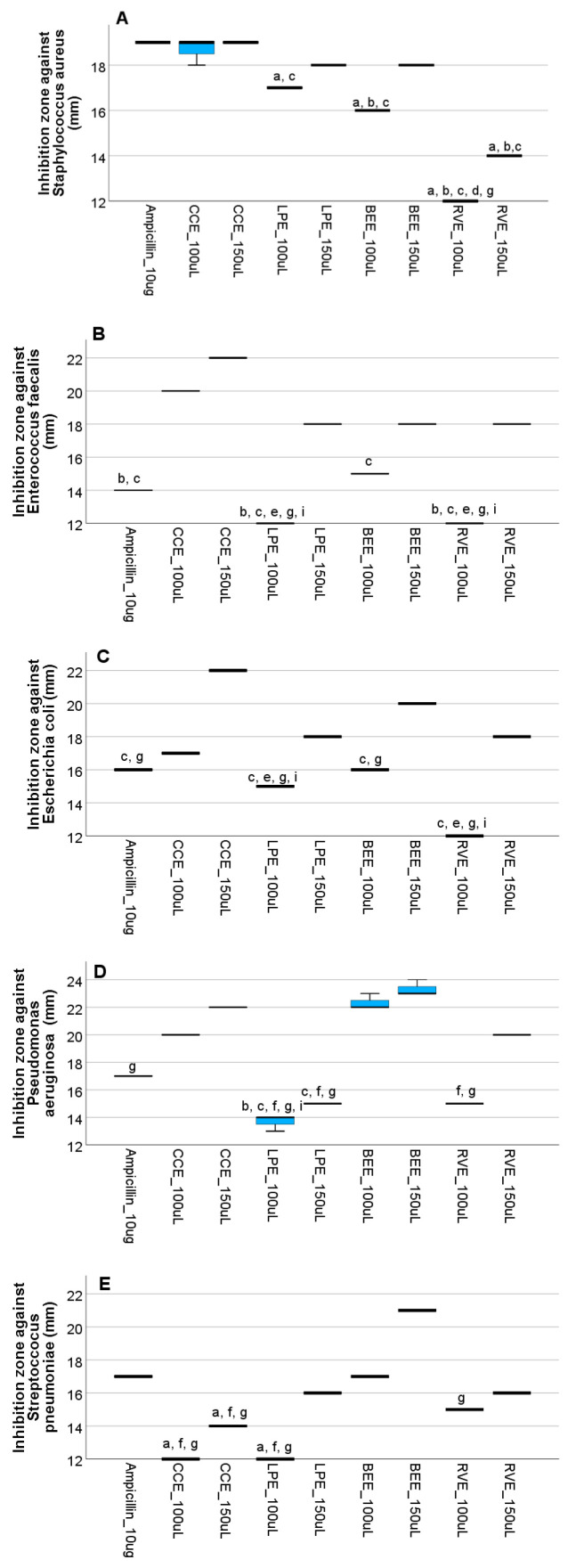
Antimicrobial activity of *Boletus edulis*, *Cantharellus cibarius*, *Lactarius piperatus*, and *Russula virescens* water extracts against *Staphylococcus aureus* (**A**), *Enterococcus faecalis* (**B**), *Escherichia coli* (**C**), *Pseudomonas aeruginosa* (**D**), and *Streptococcus pneumoniae* (**E**). The boxplots represent the median values (midline line) and the first and third quartiles (the extreme lines), where a has *p* < 0.05, versus the Ampicillin 10 ug; b has *p* < 0.05, versus CCE 100 μL; c has *p* < 0.05, versus CCE 150 μL; d has *p* < 0.05, versus LPE 100 μL; e has *p* < 0.05, versus LPE 150 μL; f has *p* < 0.05, versus BEE 100 μL; g has *p* < 0.05, versus BEE 150 μL; and i has *p* < 0.05, versus RVE 150 μL. The solutions’ concentration in phenolics in this test was 175 µg/mL for BEE, 84 µg/mL for CCE, 189 µg/mL for LPE and 97 µg/mL for RVE. The corresponding doses applied per well for the 100 µL volume were 17.5 µg for BEE, 8.4 µg for CCE, 18.9 µg for LPE and 9.7 µg for RVE, while for the 150 µL volume, the doses were 26.3 µg for BEE, 12.7 µg for CCE, 28.3 µg for LPE, and 14.5 µg for RVE.

**Figure 7:**
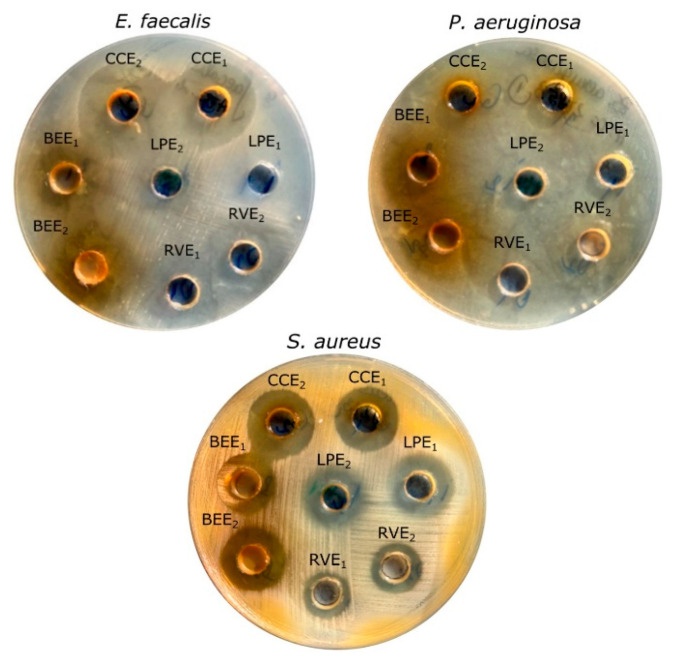
Antibacterial activity as determined using the agar diffusion method, illustrated against *Enterococcus faecalis*, *Pseudomonas aeruginosa*, and *Staphylococcus aureus*. Each notation corresponds to the mushroom extracts (BEE, CCE, LPE, and RVE), while each number corresponds to the applied volume of the sample (1–100 µL, 2–150 µL). The solutions’ concentration in phenolics in this test was 175 µg/mL for BEE, 84 µg/mL for CCE, 189 µg/mL for LPE and 97 µg/mL for RVE. The corresponding doses applied per well for the 100 µL volume were 17.5 µg for BEE, 8.4 µg for CCE, 18.9 µg for LPE and 9.7 µg for RVE, while for the 150 µL volume, the doses were 26.3 µg for BEE, 12.7 µg for CCE, 28.3 µg for LPE and 14.5 µg for RVE.

The antimicrobial activity of the investigated mushroom extracts against selected microorganisms is presented in Figure 6 and Figure 7, and Table S1. In the agar well diffusion assay, the working concentrations were 175 µg phenolics/mL for BEE, 84 µg phenolics/mL for CCE, 189 µg phenolics/mL for LPE, and 97 µg phenolics/mL for RVE. At a loading volume of 100 µL, the amounts applied per well corresponded to 17.5 µg phenolics (BEE), 8.4 µg phenolics (CCE), 18.9 µg phenolics (LPE), and 9.7 µg phenolics (RVE). When the volume was increased to 150 µL, the respective doses were 26.3 µg phenolics, 12.7 µg phenolics, 28.3 µg phenolics, and 14.5 µg phenolics per well. In general, the mushroom extracts showed promising antimicrobial activities. Among them, the CCE 150 μL showed the strongest antimicrobial activity against almost all investigated microorganisms, except the Gram-positive *S. pneumoniae*, where the MIC was lower than that of the control ampicillin. Statistically higher antimicrobial activity as compared to ampicillin was observed for the CCE 150 μL extract when tested against *E. faecalis* and *E. coli*. When tested against *S. aureus* and *P. aeruginosa*, the CCE 150 μL had the same efficacy as ampicillin, with no statistical difference observed. Further, for all mushroom extracts, a dose-dependent response was observed, with higher antimicrobial activity at 150 μL concentration. Among extracts, BEE 150 μL also had statistically significantly higher antimicrobial activity when compared to ampicillin when tested against *P. aeruginosa* and *E. coli*.

### 3.5. Antibacterial Activity of Mushroom Extracts Assessed by Time-Kill Assay

**Figure 8:**
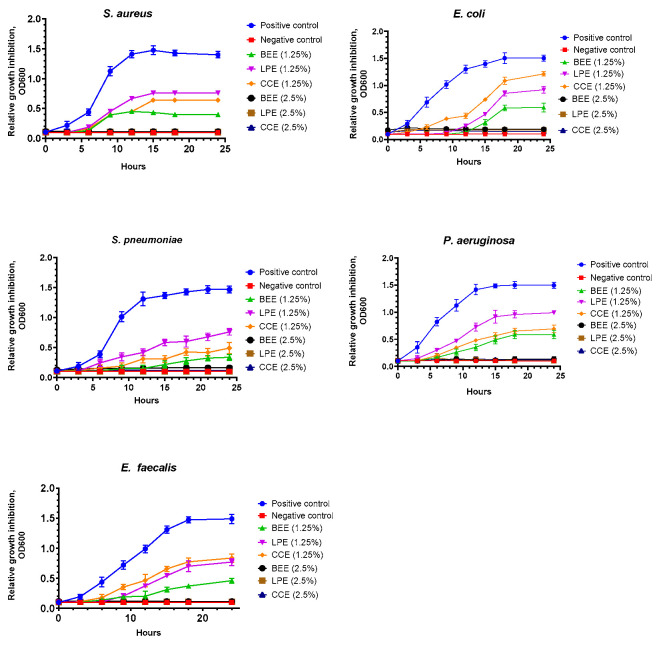
Time-kill curves showing the antibacterial activity of mushroom extracts at 1.25% and 2.5% (*v*/*v*) against selected bacterial strains over a 24 h incubation period. Bacterial growth was monitored by measuring optical density at 600 nm (OD_600_) at regular intervals. Values were normalized to the positive control (bacterial growth in broth without extract) and are expressed as relative bacterial growth (mean ± SD, *n* = 3). BEE: extract from *Boletus edulis*; LPE: extract from *Lactarius piperatus*; CCE: extract from *Cantharellus cibarius*. Positive control: bacterial suspension in Mueller–Hinton broth; negative control: broth without bacterial inoculum. For BEE, the stock extract (350.5 µg phenolics/mL) was serially diluted in DMSO to working concentrations of 175.2, 87.6, 35.0, 17.5, 8.8, and 4.4 µg phenolics/mL, corresponding to 50%, 25%, 10%, 5%, 2.5%, and 1.25% *v*/*v* of the stock solution, respectively. For CCE, the stock extract (168.9 µg phenolics/mL) was serially diluted in DMSO to working concentrations of 84.4, 42.2, 16.9, 8.4, 4.2, and 2.1 µg phenolics/mL, corresponding to 50%, 25%, 10%, 5%, 2.5%, and 1.25% *v*/*v* of the stock solution, respectively. For LPE, the stock extract (377.8 µg phenolics/mL) was serially diluted in DMSO to working concentrations of 188.9, 94.5, 37.8, 18.9, 9.4, and 4.2 µg phenolics/mL, corresponding to 50%, 25%, 10%, 5%, 2.5%, and 1.25% *v*/*v* of the stock solution, respectively.

The time-kill assay revealed a concentration-dependent antibacterial effect for all tested mushroom extracts (BEE, LPE, CCE) across the five bacterial strains. For this assay, BEE (350.5 µg phenolics/mL) was diluted to yield working concentrations of 175.2, 87.6, 35.0, 17.5, 8.8, and 4.4 µg phenolics/mL, representing 50%, 25%, 10%, 5%, 2.5%, and 1.25% (*v*/*v*) of the stock solution. Similarly, CCE (169.9 µg phenolics/mL) was diluted to 84.4, 42.2, 16.9, 8.4, 4.2, and 2.1 µg phenolics/mL, while LPE (377.8 µg phenolics/mL) was diluted to 188.9, 94.5, 37.8, 18.9, 9.4, and 4.2 µg phenolics/mL, corresponding to the same dilution levels. Figure 8 presents the relative bacterial growth over 24 h at 1.25% and 2.5% (*v*/*v*) extract concentrations, normalized to positive control. At a concentration of 2.5%, all three extracts completely inhibited bacterial growth across all tested strains, as indicated by consistently low OD_600_ values throughout the incubation. Thus, the growth of *S. aureus* was significantly inhibited by all mushroom extracts at 2.5%, with BEE and CCE showing the most pronounced activity. Notably, BEE at 2.5% nearly suppressed bacterial proliferation throughout the incubation period, approaching the performance of the positive control (ampicillin), suggesting potent bacteriostatic or potentially bactericidal effects. *E. coli* showed substantial sensitivity to BEE and CCE at 2.5%, with growth inhibition curves remaining low and stable. In contrast, treatment with 1.25% extract resulted in partial inhibition, with variable responses depending on the bacterial species and the type of extract. *L. piperatus* and RVE extracts were less effective, particularly at the lower concentration (1.25%), indicating weaker activity or partial resistance. Further, the results indicate a concentration-dependent antibacterial effect of the mushroom extracts, with greater efficacy observed against Gram-positive strains. Among the three mushroom extracts, BEE consistently showed the strongest antibacterial activity across the tested bacterial strains, followed by CCE, while LPE exhibited the weakest effect.

## 4. Discussion

One of the biggest issues facing modern medicine is antibiotic resistance. Both industrialized and underdeveloped countries are at considerable risk of dealing with the increasing number of multidrug-resistant bacterial species. The discovery or development of novel medicinal chemicals is necessary to address this worldwide problem [31,32]. Mushrooms are known sources of phytochemically active substances, including compounds with antimicrobial activities [33], such as phenolic compounds like phenolic acids, flavonoids, tannins, stilbenes, coumarins [34]. The present study assessed the phenolic profiles, antioxidant potential, antimicrobial, and antibacterial activity of water extracts from four wild mushroom species commonly found in temperate deciduous forests in Romania, namely *B. edulis*, *C. cibarius*, *L. piperatus*, and *R. virescens*.

Among the four species, *L. piperatus* exhibited the highest total phenolic content (TPC) and total flavonoid content (TFC), suggesting a rich composition of phenolic compounds. The results of this study had, in general, lower values than those reported for the same mushrooms because the present study results were expressed on a fresh weight basis. Fresh mushrooms were used to avoid potential degradation, oxidation, or transformation of thermolabile and volatile bioactive compounds that can occur during drying processes. Additionally, analysis on a fresh weight basis was chosen to provide an accurate representation of the mushrooms’ biochemical composition in their native physiological state. Further, due to the limited availability of literature data, these data were compared with reported values, despite those being based on dry weight measurements. This comparison was considered necessary to provide context and relevance to our results, acknowledging the differences in sample preparation while still allowing for a general assessment of phenolics and flavonoids total content. Thus, for the same type of aqueous extract, Fogorasi et al. [35] found higher TPC (373 mg/GAE 100 g dw versus 60 mg GAE/100 g fw) in the case of BEE, (790 mg/GAE 100 g dw versus 38 mg GAE/100 g fw) for CEE [11]. In case of LPE extract, Fogorasi et al. [35] reported lower TPC (113 mg/GAE 100 g dw versus 201 mg GAE/100 g fw), probably due to the solvent extraction, which was ethanolic in this case [35]. Also, RVE had higher TPC (36 mg/GAE 100 g dw versus 123 mg GAE/100 g fw) as reported by Srikram et al. [36]. Further, the results of total flavonoids continued the same trend as total phenolics and were in some cases, higher or lower than reported data. Thus, according to Srikram et al. [36], RVE extract had approximately 5 times higher TFC (5.37 mg/100g fw versus 1.16 mg/100g fw). The TFC for BEE was approximately 12 times lower (5.90 mg/100g fw versus 70.81 mg/100g dw), approximately two times lower for LPE (6.16 mg/100g fw versus 12.52 mg/100g dw), and approximately 4 times lower for CCE (5.85 mg/100g fw versus 20.53 mg/100g dw) as reported by Fogorasi et al. (2020) [35]. These differences can also be explained by other factors, like geographical location, harvesting period, storage conditions, extraction procedure, and the solvent used, which are the most well-known factors to induce variations.

The same approach can be associated with DPPH and ABTS antioxidant activities. For example, the DPPH radical scavenging capacity of BEE was approximately 1.3 lower than the one reported by Vamanu et al. [37] who investigated the antioxidant capacity and the correlation with major phenolics of *B. edulis* extracted using ethanol, methanol, hot water, and cold-water solvent extractions [37]. In this specific case, the slightly lower antioxidant capacity can be explained by the use of the lyophilization step prior extraction procedure. In a different study, the ABTS antioxidant capacity of BEE was found to be approximately 1.2 times higher (64 μM TE versus 52 μM TE) [38]. The differences can be explained again by the different extraction solvents and by the different climate and growing conditions of Spain compared to Romania. As a result, these analyses are difficult to compare due to inconsistent findings and non-uniform methodological frameworks.

Further, in the present study, despite its lower TPC (60 mg GAE/100 g DW), *B. edulis* demonstrated the highest antioxidant activity, both in terms of DPPH inhibition (45%) and ABTS radical scavenging capacity (64 µM Trolox/g DW). Previous studies have shown that antioxidant activity does not always correlate linearly with total phenolic content, particularly in complex natural matrices like mushrooms, where synergistic effects may differently enhance free radical scavenging but also due to differences in the specific composition and bioavailability of individual phenolic compounds [21,39].

Even though the qualitative analysis did not show a direct relation with antioxidant activity, HPLC analysis evidenced that BEE had the highest total concentration of identified phenolic compounds (351 μg/mL), including high levels of 2,3-dihydroxybenzoic acid (56 μg/mL), gallic acid (63 μg/mL), and 3-dihydroxybenzoic acid (60 μg/mL). Fogorasi et al. (2018) found that *B*. *edulis* is characterized by relatively high contents of 4-hydroxybenzoic acid (210 mg/100 g fw), cinnamic acid (169 mg/100 g fw), catechin (146 mg/100 g fw), and protocatechuic acid (44 mg/100 g fw) [40]. In *C*. *cibarius*, the main phenolics include 5-feruloylquinic (55 mg/100 g fw) and 3,5-dicaffeoylquinic acids (54 mg/100 g fw), followed by 4-hydroxybenzoic (16 mg/100 g fw) in smaller amounts [40]. *L. piperatus* exhibits notable concentrations of 4-feruloylquinic (88 mg/100 g fw), 3,5-dicaffeoylquinic (61 mg/100 g fw), and 3-feruloylquinic acids (67 mg/100 g fw), along with moderate levels of 4-hydroxybenzoic (43 mg/100 g fw) [40]. The variation in phenolic profile fingerprints and concentrations can be attributed to the differences in growth and environmental conditions, such as substrate composition, temperature, humidity, and light exposure, which can influence phenolic biosynthesis. Variations in geographical origin, mycorrhizal association, and developmental stage may also contribute to the accumulation of secondary metabolites [41,42].

The identified hydroxybenzoic acids are, as expected, known for strong radical scavenging activities [43]. These compounds possess multiple hydroxyl groups that can effectively donate hydrogen atoms to neutralize free radicals, which likely explains the strong DPPH and ABTS activities observed for BEE [44]. In contrast, LPE, which had the highest TPC (201 mg GAE/100 g DW) and total phenolics via HPLC (378 μg/mL), showed weaker antioxidant activity (DPPH: 25%; ABTS: 31 µM Trolox/g DW). These results may also be attributed to the type and reactivity of phenolics present. While LPE had a high level of 3-dihydroxybenzoic acid (170 μg/mL), it contained lower concentrations of potent antioxidants like gentisic acid (1.2 μg/mL) and gallic acid (49 μg/mL) compared to BEE. Furthermore, the presence of other compounds like proteins, lipids, and polysaccharides, as evidenced before using FTIR analysis, may also interfere with the antioxidant capacity of this extract, strengthening that TPC alone is not a sufficient predictor of antioxidant capacity, as previously discussed [45,46]. Next, RVE extract exhibited intermediate total phenolic levels and lower antioxidant activity, containing moderate amounts of 3-dihydroxybenzoic acid (73 μg/mL) and 2,3-dihydroxybenzoic acid (48 μg/mL), the lowest levels of gallic acid (12 μg/mL) and (0.07 μg/mL), explaining its poor radical scavenging. These findings align with literature suggesting that the presence of highly hydroxylated benzoic acids, especially gallic and gentisic acids, is a key contributor to antioxidant effectiveness [47,48].

The antimicrobial activity of mushroom extracts was assessed against several Gram-positive and Gram-negative bacterial strains. All extracts exhibited varying degrees of inhibition, with the highest activities recorded for CCE 150 μL and BEE 150 μL, particularly against *S. aureus*, *E. coli*, and *P. aeruginosa*. Considering phenolic composition, it is assumed that phenolic acids, due to their partially lipophilic nature, pass through the cell membrane by passive diffusion and cause an increase in membrane permeability. They possibly reduce the intracellular pH and induce protein denaturation [49,50,51]. Moreover, phenolic acids may disrupt vital enzymatic pathways, sequester metal ions essential for microbial growth, and induce oxidative stress by promoting the formation of reactive oxygen species (ROS) [52,53], contributing to their broad-spectrum antimicrobial activity. Among the possibly responsible phenolic acids quantified in the extracts, it can be observed that BEE and CEE had approximately the same quantities of *trans*-cinnamic acid (24 μg/mL versus 26 μg/mL), even though their percentage was different, as reported to the total polyphenolic compounds. Pearson correlation analysis (Table S3) also evidentiated a strong positive correlation of *trans*-cinnamic acid with antimicrobial activity against *S. aureus* at 100 μL (*r* = 0.919, *p* < 0.001) and 150 μL (*r* = 0.916, *p* < 0.001), and *E. coli* (*r* = 0.832, *p* < 0.001). As previously reported, cinnamic acid and its derivatives, whether extracted from plant sources or chemically synthesized, have been reported to exhibit antimicrobial activity against a wide range of microorganisms [54,55]. *C. cibarius* 150 μl also showed pronounced antimicrobial effects, especially against *E. faecalis* (22.2 mm) and *E. coli* (22.1 mm). Although the total phenolic concentration in CCE was lower than in BEE and LPE, CCE, besides trans cinnamic acid it still contained appreciable levels of gallic acid (41.59 μg/mL) and 3-dihydroxybenzoic acid (37 μg/mL), which likely contribute to its antibacterial potential. According to Pearson correlation analysis (Table S3), a strong positive correlation was also present for gallic acid against *S. aureus* at 100 μL (*r* = 0.701, *p* < 0.011) and 150 μL (*r* = 0.821, *p* < 0.001), and E.coli (*r* = 0.800, *p* < 0.002). It was previously reported that gallic acid possesses strong antimicrobial activity against Gram-negative bacteria like *E. coli*, *Salmonella* sp., and *Listeria* sp. [56,57,58] while 3-dihydroxybenzoic acid against *E. coli*, *P. aeruginosa*, *S. aureus*, *B. subtilis*, *S. enteritidis*, and *C. albicans* [44]. *L. piperatus*, despite its highest total phenolic content by HPLC (378 μg/mL) and TPC, showed moderate antimicrobial activity, particularly in LPE 100 μL. Interestingly, LPE 150μL demonstrated stronger inhibition zones (up to 18.2 mm) but only for *E. coli*, suggesting that the different efficacy of the mushroom extract can be due to the individual phenolic compounds’ concentration in the extract. Specifically, the high concentration of 3-dihydroxybenzoic acid in LPE (170 μg/mL) can affect the synergistic effect of the other phenolic compounds, accordingly, reducing the overall efficacy of the mixture. These results are supported by Pearson analysis, which indicated a negative correlation of 3-dihydroxybenzoic acid with antimicrobial activity against *P. aeruginosa* at 100 μL (*r* = −0.769, *p* < 0.003) and 150 μL (*r* = −0.971, *p* < 0.001), and *E. coli* at 150 μL (*r* = −0.695, *p* < 0.012). These results underscore the importance of carefully addressing the synergistic interactions between compounds and their concentrations, as even slight variations can significantly impact efficacy. Once the optimal combination is identified, the use of standardized extracts is recommended to ensure consistency, reproducibility, and therapeutic reliability.

Based on preliminary antimicrobial results, the three samples showing promising initial antimicrobial activity (BEE, LPE, and CCE) were selected for more detailed antibacterial testing. Further, the results of the antibacterial activity test demonstrated a dose-dependent inhibitory effect, with the 2.5% concentration of extracts generally producing stronger bacterial growth suppression than the 1.25% concentration. Notably, Gram-positive bacteria (*S. aureus*, *E. faecalis*, and *S. pneumoniae*) were generally more sensitive to the extracts, particularly at the lower concentration of 1.25%, compared to Gram-negative strains (*E. coli* and *P. aeruginosa*), which exhibited greater resistance. Firstly, these differences may be attributed to the distinct phytochemical profiles of the extracts, including the presence and relative abundance of phenolic compounds. Accordingly, it is already known that plant polyphenols exert their antibacterial effects through multiple mechanisms, including interactions with bacterial proteins and cell walls [59], disruption of cytoplasmic functions and membrane permeability [60], inhibition of energy metabolism [61], induction of DNA damage [62], and suppression of nucleic acid synthesis in bacterial cells [59]. Among all mechanisms, membrane disruption is one of the most prominent [63]. This effect is largely attributed to the hydroxyl (–OH) groups in phenolic compounds, which can interact with bacterial membranes through hydrogen bonding interactions [53,63].The number and position of these hydroxyl groups play a critical role in determining the antibacterial potency of polyphenols [64]. Moreover, their lipophilic nature enhances antimicrobial activity, likely due to increased affinity for and interaction with bacterial cell membranes [65]. This membrane penetration leads to irreversible structural damage, leakage of intracellular contents, and ultimately cell death [66]. Thus, as previously mentioned, the higher susceptibility of Gram-positive bacteria to the extracts is mostly because of the structural differences in the bacterial cell wall, as Gram-negative bacteria possess an outer membrane that can act as a barrier to bioactive compounds [63]. Across all tested strains, BEE and CCE consistently demonstrated the highest antibacterial efficacy, especially at 2.5% concentration. The Pearson correlation (Table S3) also evidenced a generally negative correlation of lower extract concentrations and a positive correlation of higher concentrations as a general trend. These findings support the results of the agar diffusion assays and align well with the HPLC-MS data, which showed higher concentrations of trans cinnamic, gallic acid, 2,3-dihydroxybenzoic acid, and gentisic acid in BEE and CCEs. These phenolic acids are known for disrupting microbial cell walls, inhibiting metabolic enzymes, and generating oxidative stress [44,58,67].

The limitations of the present study involve the evaluation of the antimicrobial activity of only three mushroom extracts, which may limit the generalizability of the findings. Additionally, the analysis was restricted to aqueous extracts, excluding the potential influence of other solvent systems that may extract different classes of bioactive compounds. The study did not include optimization or validation of the extraction process, and secondary extractions were not performed to confirm complete recovery of phenolic compounds. Therefore, variations in extraction efficiency among samples cannot be excluded and should be addressed in future comparative studies using TPC and antioxidant activity as optimization indicators. In addition, although a positive correlation between certain phenolic constituents and antioxidant activity was observed, possible interference from polysaccharides, proteins, and other non-phenolic compounds was not excluded. Individual phenolic monomers were not isolated or tested separately for antibacterial activity due to the exploratory scope of this study and limited extract quantities. These aspects should be addressed in future work through fractionation experiments and targeted bioassays to clarify the specific relationships between individual phenolic components and biological activity. Furthermore, assays on drug-resistant bacterial strains and cytotoxicity evaluations were not performed, and solvent comparison (e.g., ethanol vs. methanol) was not assessed. Further research should explore extracts obtained using a variety of solvents (e.g., ethanol, methanol, acetone) to better assess the full spectrum of antimicrobial and antibacterial constituents. Comparative studies are also recommended to evaluate the potential synergistic effects of these extracts when combined with conventional antibiotics, which could enhance their clinical relevance and therapeutic potential.

Finally, exploring potential synergistic interactions between these phenolic compounds either as a standardized mixture or in combination with conventional antibiotics may lead to promising therapeutic strategies to combat bacterial resistance. Such combinations, once the right balance between compounds should be discovered could act on multiple cellular targets simultaneously, enhancing antimicrobial efficacy and potentially leading to the development of more effective, broad-spectrum treatments.

## 5. Conclusions

This study highlights the significant bioactive potential of wild mushroom extracts, particularly those of *B. edulis* and *C. cibarius*, through a comprehensive evaluation of their phenolic profiles, antioxidant activities, and antimicrobial effects. HPLC-DAD-ESI(+)MS analysis revealed that trans-cinnamic, hydroxybenzoic acids—especially gallic acid, 2,3-dihydroxybenzoic acid, and gentisic acid—were predominant in BEE and CCE. Among the phenolic compounds, 2-dihydroxybenzoic acid, 2,3-dihydroxybenzoic acid, p-anisaldehyde, and gentisic acid showed positive correlations with both DPPH and ABTS antioxidant activities. Moreover, these extracts demonstrated dose-dependent antibacterial activity, with BEE and CCE showing significant growth inhibition against both Gram-positive and Gram-negative bacterial strains, including *S. aureus*, *P. aeruginosa*, and *E. coli*. The observed antimicrobial efficacy supports the hypothesis that phenolic acids not only act as radical scavengers but also disrupt microbial viability. Taken together, the findings underscore the potential of certain wild edible mushrooms as natural sources of multifunctional bioactive compounds. These species offer promising applications in nutraceuticals, functional foods, and alternative antimicrobial formulations, particularly in the context of rising antibiotic resistance. Further research should focus on compound isolation, mechanistic studies, and in vivo validations to fully explore their therapeutic relevance.

## Figures and Tables

**Table 1:** Total polyphenols content, total flavonoids content, and antioxidant activity of mushroom water extracts.

Mushroom Variety	TPC (mg GAE/100 g FW)	TFC (mg QE/100 g FW)	DPPH (%)	ABTS (%)
*Boletus edulis* (BEE)	59.64 ± 0.54 ^b^	5.90 ± 0.07 ^b^	44.73 ± 0.67 ^c^	63.99 ± 0.52 ^c^
*Lactarius piperatus* (LPE)	200.84 ± 0.90 ^d^	6.16 ± 0.12 ^c^	25.37 ± 0.20 ^b^	30.77 ± 0.55 ^a^
*Russula virescens* (RVE)	123.22 ± 0.82 ^c^	5.37 ± 0.03 ^a^	20.99 ± 0.08 ^a^	38.84 ± 0.61 ^b^
*Cantharellus cibarius* (CCE)	37.60 ± 0.84 ^a^	5.85 ± 0.04 ^b^	21.48 ± 0.20 ^a^	30.50 ± 0.33 ^a^

Values are presented as means of triplicate measurements ± standard deviation (SD). Data were analyzed by one-way ANOVA followed by Tukey’s post hoc test. Within each column, different letters indicate statistically significant differences at *p* < 0.005.

**Table 2:** Tentative identification of phenolic compounds and their concentration in mushroom water extracts (μg/mL).

Peak No.	R_t_ (min)	λ_max_ (nm)	[M+H]^+^ (m/z)	Compound	*Boletus* *edulis* Extract (BEE)	*Cantharellus cibaris* Extract (CCE)	*Lactarius* *piperatus* Extract (LPE)	*Russula* *virescens* Extract (RVE)
1	2.96	270	139	2-Dihydroxyben zoic acid ^1^	71.21 ± 0.31 ^d^	32.46 ± 0.83 ^b^	52.47 ± 0.83 ^c^	19.85 ± 0.14 ^a^
2	3.69	270	155	2,3-Dihydroxyben zoic acid ^1^	56.16 ± 0.65 ^d^	7.33 ± 0.03 ^a^	20.14 ± 0.08 ^b^	48.16 ± 0.49 ^c^
3	3.84	270	139	3-Dihydroxyben zoic acid ^1^	59.97 ± 0.55 ^b^	37.01 ± 0.30 ^a^	170.07 ± 2.10 ^d^	73.2 ± 0.91 ^c^
4	4.51	275	171	Gallic acid ^1^	63.39 ± 1.85 ^d^	41.59 ± 0.23 ^b^	49.44 ± 0.25 ^c^	11.55 ± 0.13 ^a^
5	5.27	260	137	*p*-Anisaldehyde ^1^	24.07 ± 1.08 ^d^	5.01 ± 0.01 ^b^	17.50 ± 1.32 ^c^	2.48 ± 0.01 ^a^
6	9.31	280	317	Protocatechuic acid-glucoside ^1^	20.71 ± 0.55 ^d^	5.16 ± 0.04 ^a^	19.58 ± 0.62 ^c^	10.75 ± 0.14 ^b^
7	9.72	280	155	Protocatechuic ^1^ acid	6.17 ± 0.52 ^b^	2.03 ± 0.03 ^a^	11.46 ± 0.12 ^c^	6.43 ± 0.36 ^b^
8	10.46	280	155	Gentisic acid ^1^	14.02 ± 0.28 ^d^	4.83 ± 0.12 ^c^	1.20 ± 0.02 ^b^	0.07 ± 0.00 ^a^
9	10.96	360.260	451.319	Myricetin-arabinoside ^2^	2.93 ± 0.29	1.74 ± 0.06	2.29 ± 0.08	n.d.
10	13.34	340.270	433	Apigenin-glucoside ^3^	2.48 ± 0.03 ^a^	2.55 ± 0.08 ^a^	6.56 ± 0.37 ^b^	2.59 ± 0.06 ^a^
11	14.53	360.260	481.319	Myricetin-glucoside ^2^	2.59 ± 0.16	1.81 ± 0.02	n.d.	n.d.
12	14.79	360.255	627.303	Quercetin-diglucoside ^2^	2.44 ± 0.11	1.77 ± 0.02	n.d.	n.d.
13	26.61	290	149	*trans*-Cinnamic acid ^4^	24.39 ± 1.35 ^b^	25.67 ± 0.20 ^b,c^	27.09 ± 0.14 ^c^	18.54 ± 0.14 ^a^
Total phenolics					350.51 ± 7.54	168.95 ± 1.86	377.80 ± 5.91	193.65 ± 3.39

^1^ Compounds belonging to hydroxybenzoic acid subclass. ^2^ Compounds belonging to flavonol subclass. ^3^ Compounds belonging to flavone subclass. ^4^ Compounds belonging to hydroxycinnamic acid subclass. Data were analyzed by one-way ANOVA followed by Tukey’s post hoc test. Within each row, different letters indicate statistically significant differences at *p* < 0.05. n.d. – not detected

## Data Availability

The original contributions presented in this study are included in the article/Supplementary Material. Further inquiries can be directed to the corresponding author.

## Supplementary Materials

The following supporting information can be downloaded at: https://www.mdpi.com/article/10.3390/life15111653/s1, Table S1: Inhibition zones induced by mushroom extracts against selected bacterial strains, expressed as diameter length in mm, as average and standard deviation (*n* = 3). For ampicillin (positive control), a 10 μg/disk concentration was used. DMSO was used as a negative control. Each notation corresponds to the mushroom extracts (BEE, CCE, LPE, and RVE) of 100 µL and 150 µL. The concentration of solution used in this test was 175 µg/mL for BEE, 84 µg/mL for CCE, 189 µg/mL for LPE, and 97 µg/mL for RVE. The corresponding doses applied per well for the 100 µL volume were 17.5 µg for BEE, 8.4 µg for CCE, 18.9 µg for LPE, and 9.7 µg for RVE, while for the 150 µL volume the doses were 26.3 µg for BEE, 12.7 µg for CCE, 28.3 µg for LPE, and 14.5 µg for RVE. Table S2. Pearson correlation between total phenolic compounds, total flavonoids, antioxidant activities DPPH and ABTS, and phenolic compounds. Table S3. Pearson correlation between total phenolic compounds, total flavonoids, antioxidant activities DPPH and ABTS, and phenolic compounds, antimicrobial and antibacterial activities.
