# Supplementary material for: Phenolic Compounds with Antimicrobial Properties in Mushrooms Frequently Encountered in Temperate Deciduous Forests
Source: Life (Basel). 2025 Oct 23;15(11):1653. doi: 10.3390/life15111653 (PMC12653224; doi:10.3390/life15111653)
Supplement: Supplementary file 1 [file life-15-01653-s001.zip › life-3931270-supplementary.pdf]

# Supplementary Material

# Phenolic compounds with antimicrobial properties in mushrooms frequently encountered ~~mushrooms~~ in temperate deciduous forests

**Commented [EE1]:** ATTENTION: Title altered.

**Aida Puia [1], Stanca-Lucia Pandrea [2,3]*, Jeanine Cruceru [4], Ion Cosmin Puia [5,6]*, Veronica Sanda Chedea [7], Calina Ciont [8,9], Oana Lelia Pop [8,9], Loredana Florina Leopold [8], Floricuța Ranga [8], Adriana Cristina Urcan [10], Alexandru Nicolescu [11], Otilia Bobis [12], Corina Ioana Bocsan [4], Sebastian Armean [4], Anca Dana Buzoianu [4] and Raluca Maria Pop [4]**

[1]"Iuliu Hatieganu" University of Medicine and Pharmacy, Faculty of Medicine, Department Community Medicine, Discipline of Family Medicine, 19 Moților Street, Cluj-Napoca, Romania; aida.puia@umfcluj.ro;
[2] "Iuliu Hatieganu" University of Medicine and Pharmacy, Department 1, Discipline of Microbiology, 5 Pasteur Street, Cluj-Napoca, Romania; spandrea@umfcluj.ro
[3] Octavian Fodor" Regional Institute of Gastroenterology and Hepatology, Medical Analysis Laboratory, 19-21 Croitorilor Street, Cluj-Napoca, Romania; spandrea@umfcluj.ro
[4] Pharmacology, Toxicology and Clinical Pharmacology, Department of Morphofunctional Sciences, "Iuliu Hațieganu" University of Medicine and Pharmacy, Victor Babeș, No 8, 400012, Cluj-Napoca, Romania, crucerujeanine@gmail.com; bocsan.corina@umfcluj.ro; sebastian.armean@umfcluj.ro; abuzoianu@umfcluj.ro; raluca.pop@umfcluj.com;
[5]"Iuliu Hatieganu" University of Medicine and Pharmacy, Faculty of Medicine, Department of Surgery, 19-21 Croitorilor Street, Cluj-Napoca, Romania; [6]"Octavian Fodor" Regional Institute of Gastroenterology and Hepatology, 3rd General Surgery Clinic, 19-21 Croitorilor Street, Cluj-Napoca, Romania; cosmin.puia@umfcluj.ro
[7] Research Station for Viticulture and Enology Blaj (SCDVV Blaj), 515400 Blaj, Romania; chedeaveronica@yahoo.com
[8] Faculty of Food Science and Technology, University of Agricultural Sciences and Veterinary Medicine, 3–5 Calea Mănăștur, 400372 Cluj-Napoca, Romania; calina.ciont@usamvcluj.ro; oana.pop@usamvcluj.ro; loredana.leopold@usamvcluj.ro; florica.ranga@usamvcluj.ro
[9] Molecular Nutrition and Proteomics Laboratory, Institute of Life Sciences, University of Agricultural Sciences and Veterinary Medicine, 400372, Cluj-Napoca, Romania;
[10] Department of Microbiology and Immunology, Faculty of Animal Science and Biotechnology, University of Agricultural Sciences and Veterinary Medicine, 400372, Cluj-Napoca, Romania; adriana.urcan@usamvcluj.ro
[11] Laboratory of Chromatography, Faculty of Horticulture and Business in Rural Development, University of Agricultural Sciences and Veterinary Medicine, 400372, Cluj-Napoca, Romania; alexandru.nicolescu@usamvcluj.ro
[12] Department of Apiculture and Sericulture, Faculty of Animal Science and Biotechnology, University of Agricultural Sciences and Veterinary Medicine Cluj Napoca, 400372 Cluj-Napoca, Romania; obobis@usamvcluj.ro
* Correspondence: author: Stanca-Lucia Pandrea: spandrea@umfcluj.ro; Ion Cosmin Puia: cosmin.puia@umfcluj.ro

**Table S1.** Inhibition zones induced by mushroom extracts against selected bacterial strains, expressed as diameter length in mm, as and average and standard deviation (*n* = 3). For ampicillin (positive control), a 10 μg/disk concentration was used. DMSO was used as a negative control. Each notation corresponds to the mushroom extracts (BEE, CCE, LPE, and RVE) of 100 μL and 150 μL. The concentration of solution used in this test was 175 μg/mL for BEE, 84 μg/mL for CCE, 189 μg/mL for LPE and 97 μg/mL for RVE. The corresponding doses applied per well for the 100 μL volume were 17.5 μg for BEE, 8.4 μg for CCE, 18.9 μg for LPE and 9.7 μg for RVE, while for the 150 μL volume, the doses were 26.3 μg for BEE, 12.7 μg for CCE, 28.3 μg for LPE and 14.5 μg for RVE.

| Tested extract | *S. aureus* | *E. faecalis* | *E. coli* | *P. aeruginosa* | *S. pneumoniae* |
|---|---|---|---|---|---|
| BEE1 | 16.1 ± 0.1 | 15.2 ± 0.2 | 16.1 ± 0.1 | 22.6 ± 0.1 | 17.1 ± 0.1 |
| BEE2 | 18.1 ± 0.1 | 18.2 ± 0.1 | 20.2 ± 0.2 | 23.5 ± 0.1 | 21.1 ± 0.2 |
| CEE1 | 18.5 ± 0.5 | 20.2 ± 0.2 | 17.1 ± 0.1 | 20.2 ± 0.1 | 12.1 ± 0.1 |
| CEE2 | 19.1 ± 0.1 | 22.2 ± 0.2 | 22.1 ± 0.2 | 22.2 ± 0.2 | 14.1 ± 0.1 |
| LPE1 | 17.1 ± 0.1 | 12.2 ± 0.1 | 15.3 ± 0.1 | 13.5 ± 0.1 | 12.1 ± 0.1 |
| LPE2 | 18.1 ± 0.1 | 18.1 ± 0.2 | 18.2 ± 0.1 | 15.2 ± 0.1 | 16.1 ± 0.1 |
| RVE1 | 12.1 ± 0.1 | 12.1 ± 0.1 | 12.2 ± 0.1 | 15.2 ± 0.1 | 15.1 ± 0.1 |
| RVE2 | 14.1 ± 0.1 | 18.1 ± 0.1 | 18.1 ± 0.1 | 20.2 ± 0.1 | 16.1 ± 0.1 |
| Ampicillin | 19.0 ± 0.0 | 14.0 ± 0.0 | 16.0 ± 0.0 | 17.0 ± 0.0 | 17.0 ± 0.0 |
| DMSO | 6.0 ± 0.0 | 6.0 ± 0.0 | 6.0 ± 0.0 | 6.0 ± 0.0 | 6.0 ± 0.0 |

Table S2. Pearson correlation between total phenolic compounds, total flavonoids, antioxidant activities DPPH and ABTS (see Table 1) and phenolic compounds (see Table 2).

| r (*p*) | TPC | TFC | DPPH | ABTS | 2-Dihydroxybenzoic acid | 2,3-Dihydroxybenzoic acid | 3-Dihydroxybenzoic acid | Gallic acid | p-Anisaldehida | Protocatechuic acid-glucoside | Protocatechuic acid | Gentisic acid | Myricetin-arabinoside | Apigenin-glucoside | Myricetin-glucoside | Quercetin-diglucoside | trans-Cinamic acid |
|---|---|---|---|---|---|---|---|---|---|---|---|---|---|---|---|---|---|
| TPC | 1 | 0.313 | -0.281 | -0.386 | -0.016 | -0.052 | .955** | -0.163 | 0.124 | 0.471 | .929** | -.611* | 0.088 | .871** | .974** | .977** | 0.078 |
| | | (0.322) | (0.376) | (0.215) | (0.961) | (0.872) | (0.000) | (0.614) | (0.702) | (0.122) | (0.000) | (0.035) | (0.821) | (0.000) | (0.001) | (0.001) | (0.810) |
| TFC | 0.313 | 1 | 0.229 | -0.119 | .620* | -0.442 | 0.562 | .739** | .623* | 0.473 | 0.417 | 0.171 | 0.007 | .699* | 0.259 | 0.293 | .908** |
| | (0.322) | | (0.474) | (0.714) | (0.032) | (0.150) | (0.057) | (0.006) | (0.031) | (0.120) | (0.177) | (0.596) | (0.986) | (0.011) | (0.621) | (0.573) | (0.000) |
| DPPH | -0.281 | 0.229 | 1 | .932** | .889** | .625* | -0.125 | .749** | .865** | .713** | 0.085 | .921** | .899** | -0.186 | .970** | .988** | 0.199 |
| | (0.376) | 0.474 | | (0.000) | (0.000) | (0.030) | (0.698) | (0.005) | (0.000) | (0.009) | (0.794) | (0.000) | (0.001) | (0.563) | (0.001) | (0.000) | (0.534) |
| ABTS | -0.386 | -0.119 | .932** | 1 | .666* | .826** | -0.328 | 0.468 | .648* | 0.562 | -0.060 | .862** | .848** | -0.446 | .971** | .986** | -0.157 |
| | (0.215) | (0.714) | (0.000) | | (0.018) | (0.001) | (0.298) | (0.125) | (0.023) | (0.057) | (0.852) | (0.000) | (0.004) | (0.146) | (0.001) | (0.000) | (0.626) |
| 2-Dihydroxybenzoic acid | -0.016 | .620* | .889** | .666* | 1 | 0.289 | 0.221 | .933** | .986** | .817** | 0.328 | .780** | .959** | 0.228 | .974** | .981** | .579* |
| | (0.961) | (0.032) | (0.000) | (0.018) | | (0.362) | (0.490) | (0.000) | (0.000) | (0.001) | (0.298) | (0.003) | (0.000) | (0.476) | (0.001) | (0.001) | (0.048) |
| 2,3-Dihydroxybenzoic acid | -0.052 | -0.442 | .625* | .826** | 0.289 | 1 | -0.153 | -0.034 | 0.344 | 0.510 | 0.158 | 0.429 | .929** | -0.376 | .972** | .985** | -.594* |
| | (0.872) | (0.150) | (0.030) | (0.001) | (0.362) | | (0.635) | (0.918) | (0.273) | (0.090) | (0.625) | (0.164) | (0.000) | (0.228) | (0.001) | (0.000) | (0.042) |
| 3-Dihydroxybenzoic acid | .955** | 0.562 | -0.125 | -0.328 | 0.221 | -0.153 | 1 | 0.124 | 0.340 | .590* | .948** | -0.434 | 0.120 | .965** | .970** | .988** | 0.354 |
| | (0.000) | (0.057) | (0.698) | (0.298) | (0.490) | (0.635) | | (0.702) | (0.280) | (0.044) | (0.000) | (0.159) | (0.758) | (0.000) | (0.001) | (0.000) | (0.259) |
| Gallic acid | -0.163 | .739** | .749** | 0.468 | .933** | -0.034 | 0.124 | 1 | .878** | .591* | 0.131 | .756** | .926** | 0.219 | .980** | .984** | .791** |
| | (0.614) | (0.006) | (0.005) | (0.125) | (0.000) | (0.918) | (0.702) | | (0.000) | (0.043) | (0.686) | (0.004) | (0.000) | (0.493) | (0.001) | (0.000) | (0.002) |
| p-Anisaldehida | 0.124 | .623* | .865** | .648* | .986** | 0.344 | 0.340 | .878** | 1 | .897** | 0.459 | .697* | .924** | 0.321 | .980** | .984** | 0.537 |
| | (0.702) | (0.031) | (0.000) | (0.023) | (0.000) | (0.273) | (0.280) | (0.000) | | (0.000) | (0.134) | (0.012) | (0.000) | (0.308) | (0.001) | (0.000) | (0.072) |

| r (*p*) | TPC | TFC | DPPH | ABTS | 2-Dihydroxybenzoic acid | 2,3-Dihydroxybenzoic acid | 3-Dihydroxybenzoic acid | Gallic acid | p-Anisaldehida | Protocatechuic acid-glucoside | Protocatechuic acid | Gentisic acid | Myricetin-arabinoside | Apigenin-glucoside | Myricetin-glucoside | Quercetin-diglucoside | trans-Cinamic acid |
|---|---|---|---|---|---|---|---|---|---|---|---|---|---|---|---|---|---|
| Protocatechuic acid-glucoside | 0.471 | 0.473 | .713** | 0.562 | .817** | 0.510 | .590* | .591* | .897** | 1 | .754** | 0.403 | .837** | 0.482 | .977** | .985** | 0.275 |
| | (0.122) | (0.120) | (0.009) | (0.057) | (0.001) | (0.090) | (0.044) | (0.043) | (0.000) | | (0.005) | (0.194) | (0.005) | (0.113) | (0.001) | (0.000) | (0.387) |
| Protocatechuic acid | .929** | 0.417 | 0.085 | -0.060 | 0.328 | 0.158 | .948** | 0.131 | 0.459 | .754** | 1 | -0.282 | 0.387 | .844** | .931** | .988** | 0.174 |
| | (0.000) | (0.177) | (0.794) | (0.852) | (0.298) | (0.625) | (0.000) | (0.686) | (0.134) | (0.005) | | (0.374) | (0.304) | (0.001) | (0.007) | (0.000) | (0.589) |
| Gentisic acid | -.611* | 0.171 | .921** | .862** | .780** | 0.429 | -0.434 | .756** | .697* | 0.403 | -0.282 | 1 | .693* | -0.423 | .972** | .982** | 0.253 |
| | (0.035) | (0.596) | (0.000) | (0.000) | (0.003) | (0.164) | (0.159) | (0.004) | (0.012) | (0.194) | (0.374) | | (0.039) | (0.171) | (0.001) | (0.000) | (0.427) |
| Myricetin-arabinoside | 0.088 | 0.007 | .899** | .848** | .959** | .929** | 0.120 | .926** | .924** | .837** | 0.387 | .693* | 1 | -0.043 | .938** | .907* | -0.492 |
| | (0.821) | (0.986) | (0.001) | (0.004) | (0.000) | (0.000) | (0.758) | (0.000) | (0.000) | (0.005) | (0.304) | (0.039) | | (0.913) | (0.006) | (0.012) | (0.178) |
| Apigenin-glucoside | .871** | .699* | -0.186 | -0.446 | 0.228 | -0.376 | .965** | 0.219 | 0.321 | 0.482 | .844** | -0.423 | -0.043 | 1 | -0.647 | -0.612 | 0.540 |
| | (0.000) | (0.011) | (0.563) | (0.146) | (0.476) | (0.228) | (0.000) | (0.493) | (0.308) | (0.113) | (0.001) | (0.171) | (0.913) | | (0.165) | (0.196) | (0.070) |
| Myricetin-glucoside | .974** | 0.259 | .970** | .971** | .974** | .972** | .970** | .980** | .980** | .977** | .931** | .972** | .938** | -0.647 | 1 | .929** | -0.480 |
| | (0.001) | (0.621) | (0.001) | (0.001) | (0.001) | (0.001) | (0.001) | (0.001) | (0.001) | (0.001) | (0.007) | (0.001) | (0.006) | (0.165) | | (0.007) | (0.336) |
| Quercetin-diglucoside | .977** | 0.293 | .988** | .986** | .981** | .985** | .988** | .984** | .984** | .985** | .988** | .982** | .907* | -0.612 | .929** | 1 | -0.620 |
| | (0.001) | (0.573) | (0.000) | (0.000) | (0.001) | (0.000) | (0.000) | (0.000) | (0.000) | (0.000) | (0.000) | (0.000) | (0.012) | (0.196) | (0.007) | | (0.189) |
| trans-Cinamic acid | 0.078 | .908** | 0.199 | -0.157 | .579* | -.594* | 0.354 | .791** | 0.537 | 0.275 | 0.174 | 0.253 | -0.492 | 0.540 | -0.480 | -0.620 | 1 |
| | (0.810) | (0.000) | (0.534) | (0.626) | (0.048) | (0.042) | (0.259) | (0.002) | (0.072) | (0.387) | (0.589) | (0.427) | (0.178) | (0.070) | (0.336) | (0.189) | |

Correlations were classified as very strong when r ranged from 0.90 to 0.99 or from –0.99 to –0.90, strong when r ranged from 0.70 to 0.89 or from –0.89 to –0.70, and moderate when r ranged from 0.40 to 0.69 or from –0.69 to –0.40.

**Correlation is statistically significant at the $p < 0.01$ level (2-tailed).

* Correlations is statistically significant at the < 0.05 level (2-tailed).
